# Role of Farnesoid X Receptor in the Determination of Liver Transcriptome during Postnatal Maturation in Mice

**DOI:** 10.11131/2017/101308

**Published:** 2017-10-20

**Authors:** Lai Peng, Stephanie C. Piekos, Grace L. Guo, Xiao-bo Zhong

**Affiliations:** 1 Department of Pharmaceutical Sciences, School of Pharmacy, University of Connecticut, Storrs, CT 06269, USA; 2 Department of Pharmacology and Toxicology, School of Pharmacy, Rutgers University, Piscataway, NJ 08807, USA

**Keywords:** FXR, RNA-Seq, transcriptome, liver, postnatal maturation

## Abstract

The liver is a vital organ with critical functions in metabolism of various biologically useful materials, synthesis of several vital proteins, detoxification of toxic substances, and immune defense. Most liver functions are not mature at birth and many changes happen during postnatal liver development, which lead to differential vulnerabilities of the liver at different developmental stages. However, the details of what changes occur in liver after birth, at what developmental stages they occur, and molecular mechanisms in the regulation of the developmental process are not clearly known. The nuclear receptor Farnesoid X receptor (FXR) is an important transcriptional regulator in liver. Here, we used RNA-Sequencing to analyze the transcriptome of mouse liver from perinatal to adult ages in both C57BL/6 and *Fxr*^−/−^ mice. We have defined a clear timeline of functional transition from prenatal through neonatal and adolescent to adult in C57BL/6 mice. Without FXR, activation of neonatal-specific pathways was prolonged and maturation of multiple metabolic pathways was delayed. The loss of FXR also led to increased expression of 27 other transcription regulators. Our data support a conclusion that developmental transcriptome revealed significant functional transition during postnatal liver development and FXR plays an important role in control of postnatal liver maturation.

## 1. Introduction

In the adult, the liver is a mature organ with critical, well-defined roles in the maintenance of nutrient homeostasis, bile acid synthesis, the metabolism of xenobiotics and endogenous hormones, and the detoxification of exogenous compounds [1]. In the developing fetus and neonate, however, the liver acts as a hematopoietic organ with a distinct function in generating blood cells [2]. During the period of postnatal development, a transition occurs in order for the liver to gain mature metabolic activity. Significant changes in gene expression take place during this time in order to facilitate the switch from a hepatic microenvironment supportive of hematopoiesis to one that promotes a broad range of metabolic and detoxifying functions [3].

Differences in the metabolic capabilities of neonatal and adult livers are implicated in clinical therapeutic challenges. It is known that the activity of drug metabolizing enzymes changes dramatically throughout human postnatal development, resulting in pediatric pharmacokinetic and detoxification pathways being unique from adults [4]. Pediatric patients can be at a higher risk of developing drug-induced liver injuries when treated with drugs that require detoxification by the liver. On the other hand, infants can also be more resistant to liver damage when toxic drug metabolites are produced via metabolism or require higher doses [5]. Hyperbilirubinemia, or neonatal jaundice, is also common in infants during the first weeks of life. This condition is characterized by a buildup of bilirubin due to the neonatal liver’s reduced capacity for bilirubin conjugation and excretion and can lead to neurotoxicity or cellular injury [6].

While our understanding of embryonic liver development has greatly expanded over recent years, the regulation of postnatal liver development and maturation remains a relatively understudied area. There is still a need to identify key transcription factors, external environmental signals, and cellular mechanisms that promote liver growth and maturation following birth. Nuclear receptors are known to act as sensors for both endogenous and exogenous compounds that can then affect gene transcription upon their activation. Because of this, many nuclear receptors have critical roles in adult liver functions, diseases, and regeneration, and allow the organ to respond to environmental stimuli, such as drug exposure [7]. The farnesoid X receptor (FXR), encoded by the *NR1H4* gene, is highly expressed in the liver and becomes activated upon the binding of bile acids. FXR is best characterized for its role in cholesterol synthesis and bile acid homeostasis, although it is also a major regulator of hepatic triglyceride and glucose levels [8–10]. Aside from nutrient homeostasis, FXR has also appears to play a role in liver regeneration and repair, as bile acids promote liver regeneration while the deletion of FXR delays the regeneration process in rodents [11–13].

The importance of FXR in hepatic physiology and diseases has mainly been studied in the context of the fully matured adult liver and microenvironment. However, its role in liver regeneration suggests that FXR may also be a critical factor in liver growth during postnatal development. Following birth, a surge of bile acids enters the circulation as the neonate in order to facilitate the digestion of high-fat milk. The activation of FXR by these bile acids may initiate the liver’s transition from a hematopoietic organ to a fully matured metabolic organ by regulating gene expression. Bile acids have already been shown to initiate the expression of hepatic bile acid transporters and Phase I drug metabolizing enzymes via FXR in newborn mice [14, 15]. Understanding how FXR regulates the rest of the hepatic transcriptome following birth and during postnatal development may give insight to mechanisms controlling the liver’s functional switch in early life and the development of diseases, such as non-alcoholic steatohepatitis, later in life, as they may originate from epigenetic programming at the neonatal age [16].

In the current study, RNA-sequencing (RNA-seq) was used to characterize the mouse liver transcriptome at various ages throughout the perinatal stage to adulthood in both wild type C57BL/6 and FXR knockout mice with C57BL/6 background. Analysis of the data focuses on the role FXR in influencing hepatic gene expression throughout development and initiating the functional switch from a hematopoietic organ to a mature metabolic organ by comparing ontogenic differences in the presence versus the absence of FXR. The results validate FXR as a major factor in the regulation of postnatal liver maturation and provide defined ages, at which dynamic changes in the hepatic transcriptome occur.

## 2. Materials and Methods

### 2.1. Animals

The mice were bred and cared under standard conditions according to the criteria outlined in the NIH “Guide for the Care and Use of Laboratory Animals” in the Office of Animal Care Facility at the University of Connecticut. The use of these mice was approved by the University of Connecticut’s Institutional Animal Care and Use Committee. Liver samples (n=3) were collected at the following 6 ages: day −2 (gestational day 17.5), day 1 (exactly 24 hours after birth), and days 5, 20, 25, and 60 (collected at approximately 9:00 AM). These ages represent the periods of prenatal (day −2), neonatal (days 1 and 5), adolescent (days 20 and 25), and young adults (day 60). Due to potential variations caused by the estrous cycle in maturing female mice, only male livers were used in this study. The livers were immediately frozen in liquid nitrogen after removal and stored at −80°C.

### 2.2. Total RNA extraction, sequencing library construction, and RNA-Seq

Transcriptomes of C57BL/6 mouse livers at different developmental ages were previously determined [17] and ontogenic patterns of gene expression for cytochrome P450s [18], non-P450 phase I drug metabolizing enzymes [19], phase II drug metabolizing enzymes [20], transporters [21], epigenetic modifiers [22], energy metabolism genes [23], and long non-coding RNAs [24] were established. The generation and characterization of *Fxr*^−/−^ mice on a C57BL/6 background are previously described [25, 26]. Transcriptomes of *Fxr*^−/−^ mouse livers at different developmental ages were determined in this study. RNA extraction, library construction, RNA-Seq, and FASTQ data file collection were performed as previously described [18, 19, 21].

### 2.3. RNA-Seq data analysis

For comparison of C57BL/6 and *Fxr*^−/−^ samples at the 6 ages of day −2, 1, 5, 20, 25 and 60, the RNA-Seq reads from the FASTQ files of these two types of mice were mapped to the mouse reference genome (GRCm38/mm10) by Tophat 2.0.8. The output files in BAM format were analyzed by Cufflinks 2.1.1 to estimate the transcript abundance, represented as fragments per kilobase of exon per million reads mapped (FPKM).

### 2.4. Analysis of differentially expressed genes

Genes with statistically significant differential expression were defined by ANOVA using the following criteria: 1) the gene mean FPKM > 1; 2) fold change for the average FPKM of the three replicates > 1.5 between compared samples; 3) Benjamini-Hochberg adjusted p-values from t-test < 0.05.

### 2.5. Similarity of transcriptome among the liver samples at different ages

Pearson correlation coefficient-based heat map visualization of the average FPKM of the three replicates at each age were used to examine similarity of transcriptome among the liver samples at different ages.

### 2.6. Gene ontology analysis

Lists of differentially expressed genes at ages of day −2, 1, 5, 20 and 25 compared to day 60 in both C57BL/6 and *Fxr*^−/−^ mice were supplied to High-Throughput GoMiner for biological interpretation [27]. Significantly enriched GO categories were selected with a false discovery rate (FDR)<0.05. For the visualization of nuclear receptors and core transcription factors expression in liver, significant differential expressions were defined in the same way as in GO analysis.

### 2.7. Data access

The RNA-Seq data for C57BL/6 and *Fxr*^−/−^ mice are available from GEO under the accession number GSE58827 (http://www.ncbi.nlm.nih.gov/geo/query/acc.cgi?acc=GSE58827).

### 2.8. Validation of transcription profiles defined by RNA-Seq with RT-PCR

Expression of selected genes defined by RNA-Seq in both C57BL/6 and *Fxr*^−/−^ mice was validated by RT-PCR. Three wild type replicates and three *FXR*^−/−^ replicates were used for each age in RT-PCR analysis. Total RNA was converted into cDNA using Reverse Transcription kit according to the manufacturer’s protocol (Bio-Rad). PCR was performed using a CFX-96 thermocycler system (Bio-Rad, Hercules, CA, USA). To create the reaction mixture, 100 ng cDNA was added to 2X SYBR Green PCR Master Mix (Bio-Rad), with 10 μM forward primers and 10 μM reverse primers. Primer sequences for selected genes are listed in Supplemental Table S1 available online at http://www.agialpress.com/journals/nurr/2017/101308. PCR was conducted at 95°C for 3 minutes, followed by 40 cycles of 95°C for 10 seconds and 60°C for 1 minute. Results of RT-PCR for selected genes are shown in Supplemental Figure S1 available online at http://www.agialpress.com/journals/nurr/2017/101308, alongside FPKM results. The supplemental Table S2 available online at http://www.agialpress.com/journals/nurr/2017/101308 shows a strong correlation for the ten selected genes with Pearson’s correlation *r* values between 0.857 and 0.967 for C57BL/6 mice and between 0.960 and 0.999 for *Fxr*^−/−^ mice between the two quantification methods.

## 3. Results

### 3.1. FXR gene expression during postnatal liver maturation in mice

**Figure 1:**
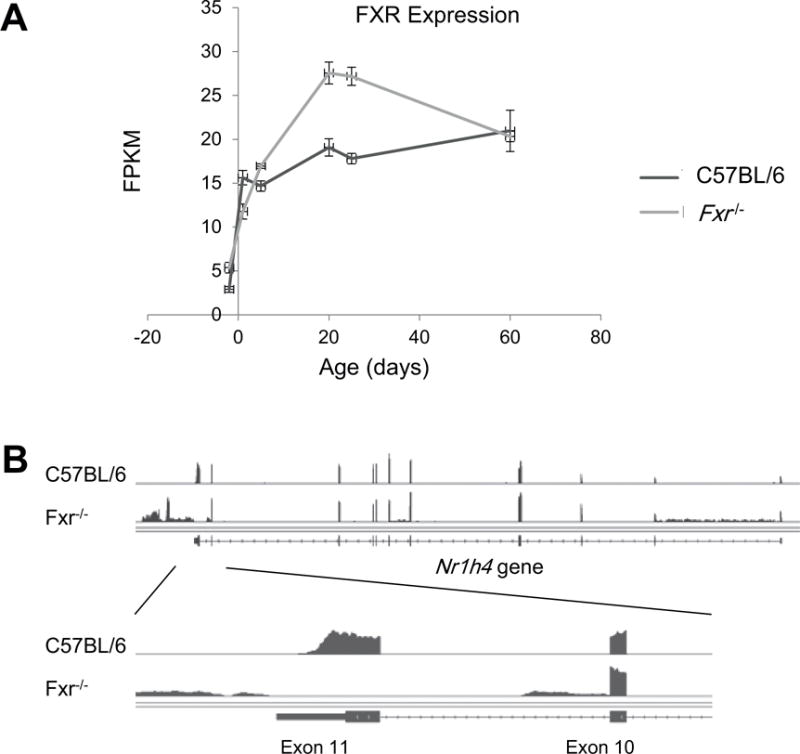
(A) mRNA Expression of FXR revealed by RNA-Seq during postnatal liver maturation. (B) Confirmation of the deletion of the last exon of the *Fxr* gene in *Fxr*^−/−^ mice revealed by RNA-Seq. Distribution of RNA-Seq reads were viewed on IGV genome browser.

Six ages from the four developmental stages were selected to analyze the liver transcriptome at day −2 (perinatal), day 1 and day 5 (neonatal), day 20 and day 25 (adolescent), and day 60 (adult). RNA-Seq generated an average 137 million (from 103 to 164 million) 100 bp paired end reads for the 36 samples from 6 different ages for C57BL/6 and *Fxr*^−/−^ mice with a mean of 86% (from 73 to 95%) reads mapped to the mouse reference genome (GRCm38/mm10). Unique mapped reads were assigned to each gene by Cufflinks to define gene expression levels represented by FPKM to 23,297 annotated genes in the mouse genome (Supplemental Table S3 available online at http://www.agialpress.com/journals/nurr/2017/101308). RNA-Seq revealed that the mRNA expression of FXR was relatively low in prenatal liver at day −2, but immediately increased at day 1 and remained consistent through postnatal maturation in C57BL/6 mice (Figure 1A). RNA-Seq also showed that FXR was transcribed in the *Fxr*^−/−^ mice, but the RNA-Seq reads for the last exon of the *Fxr* gene (exon 11) was undetectable in the *Fxr*^−/−^ mice (Figure 1B), which confirmed that the *Fxr* null mice lacked the last exon, which contains a large portion of the ligand binding/dimerization domain and the entire 3′-untranslated region [25], and thus is not functional.

### 3.2. Comparison of transcriptome of C57BL/6 and *Fxr*^−/−^ mice during postnatal liver maturation

Table 1 lists number of genes expressed at each examined age for both C57BL/6 and *Fxr*^−/−^ livers, when genes with FPKM >1 were considered as the genes expressed in liver. The expressed genes for C57BL/6 remained consistent between 10,338 at prenatal day −2 and 10,402 at neonatal day 5, but decreased about 19% in adult liver (day 60) to around 8507; whereas the expressed genes for *Fxr*^−/−^ also remained consistently between 11,577 at prenatal day −2 and 11,698 at neonatal day 5, but decreased to around 10,058 at adult day 60 (13% decreased compared to prenatal day −2 of *Fxr*^−/−^).

**Figure 2:**
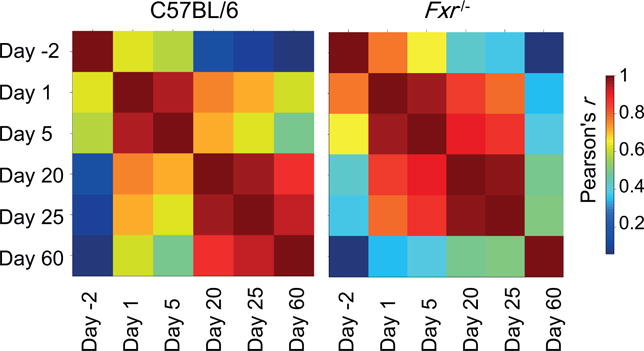
Similarity of gene expression during postnatal liver maturation in C57BL/6 and *Fxr*^−/−^ mice. Pearson correlation coefficient-based heat maps are drawn to present the similarity of gene expression profiles based on all expressed genes over 6 different ages.

The similarity of gene expression across the postnatal liver maturation was analyzed in both C57BL/6 and *Fxr*^−/−^samples with the Pearson’s correlation coefficient *r* analysis (Figure 2). The correlation heat map of the 6 developmental ages displayed clear developmental stage transition in C57BL/6 liver. But the correlation heat map for *Fxr*^−/−^samples exhibited dramatic differences in the developmental stage transition. The outstanding feature was that the difference between adolescent (day 20 and 25) and neonatal (day 1 and 5) stages was diminished, implying that the deletion of *Fxr* appears to delay the postnatal liver maturation.

### 3.3. Timeline of liver functional transition in C57BL/6 mice

**Figure 3:**
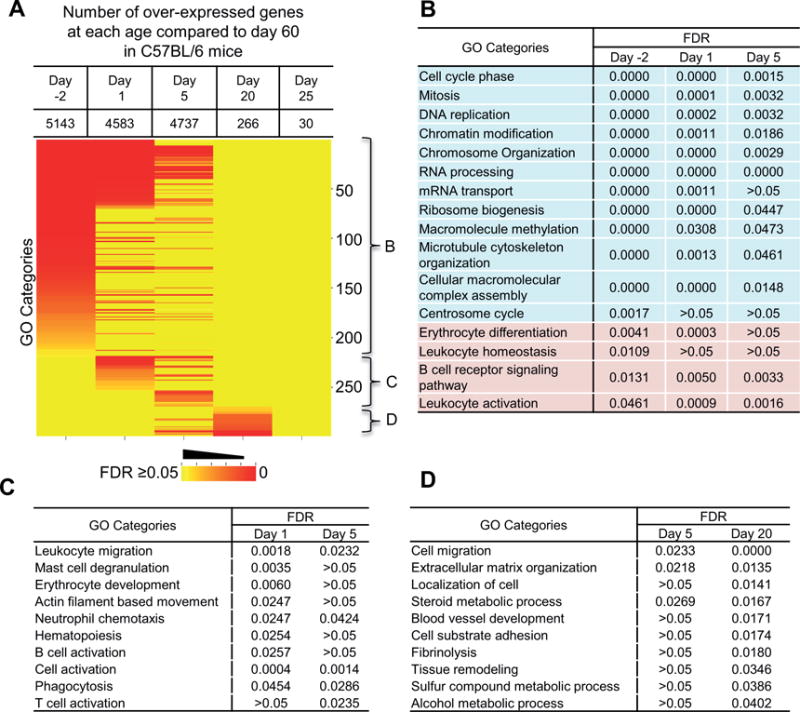
Gene ontology (GO) analysis for over-expressed genes during postnatal liver maturation compared to day 60. (A) Heat map for GO categories with significantly overexpressed genes at ages of day −2, 1, 5, 20, and 25. The color represents the false discovery rate (FDR) of each GO category. The numbers on the right are the cumulative numbers of GO categories. The three brackets B, C, and D identify groups of GO categories enriched at specific ages during development. Representative GO categories in each bracket are show in the corresponding panels of (B), (C), and (D).

Livers of 60 day-old mice were considered as mature and the transcriptome of each younger age was compared to that of 60 day-old mice for defining the timeline of liver function transition during postnatal maturation. Both significantly over-expressed and under-expressed genes were identified by ANOVA analysis. The identified genes were further used for identification of effected biological pathways by gene ontology (GO) analysis with the GoMiner tool. The enriched GO categories in the over-expressed genes at earlier ages and their significance false discovery rate (FDR) levels across the ages are shown in Figure 3A. The number of over-expressed genes at each age was listed in the top of Figure 3A. There was a clear developmental transition of enriched biological processes in these over-expressed genes as indicated by the brackets B, C and D, which correlated to different ages. GO categories in bracket B were specific to perinatal and neonatal stages, and representative GO categories in this group are shown in Figure 3B. Enriched biological processes at the prenatal and neonatal stages were mainly related to cell cycle and basic cellular processing, suggesting active cell proliferation in prenatal and neonatal livers. GO categories related to blood cell development and homeostasis are also enriched at these stages, which correspond to the hematopoietic function of perinatal liver. Typical GO categories in bracket C, which were specific to neonatal livers at day 1 and 5, are shown in Figure 3C. The major enriched pathways at this stage were related to blood cell generation and immune activation. So the neonatal liver still retained residual hematopoietic function from fetal liver. With exposure to the environment after birth, the immune system became activated. Representative GO categories enriched at day 20 are shown in Figure 3D, which are the pathways related to tissue structure organization, and a few metabolic process (alcohol and sulfur compounds). At day 25, there were no significant GO categories with gene over-expression compared to day 60.

**Figure 4:**
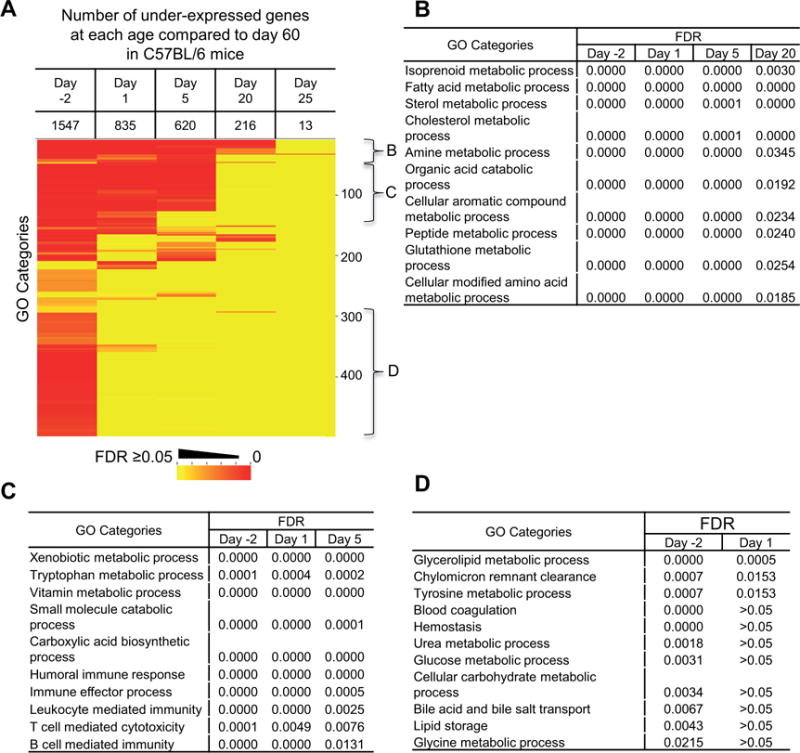
Gene ontology analysis for under-expressed genes during postnatal liver maturation compared to day 60. (A) Heat map for GO categories with significant under-expression genes at ages of day −2, 1, 5, 20, and 25. The color represents the false discovery rate of each GO category. The numbers on the right are the cumulative number of GO categories. The three brackets B, C, and D identify groups of GO categories enriched at specific ages during postnatal liver maturation. Representative GO categories in each bracket are show in the corresponding panels of (B), (C), and (D).

A similar GO analysis was done for the under-expressed genes during postnatal liver maturation (Figure 4). There were fewer under-expressed genes as the age became closer to day 60 (Figure 4A). Day 25 samples had a few significant GO categories, indicating the liver was close to mature at this age. The majority of pathways with genes under-expressed were different metabolic processes. One group matured relatively late (day 20) during postnatal maturation (see a list in Figure 4B, including metabolism of fatty acids, sterols, cholesterol, organic acids, glutathione, and amino acids). Under-expressed GO categories specific to age day −2, 1, and 5 were biological processes related to metabolism of different compounds, including xenobiotics, vitamin, tryptophan, small molecules, as well as immunity (Figure 4C). The representative GO categories that matured early during development (day 1) are listed in Figure 4D, which include pathways of critical nutrient metabolism, such as glucose, lipid, and amino acids, bile acids transport, and blood coagulation. These results revealed details of hepatic functional transition and maturation during postnatal liver maturation.

### 3.4. Timeline of liver functional transition in *Fxr*^−/−^ mice

**Figure 5:**
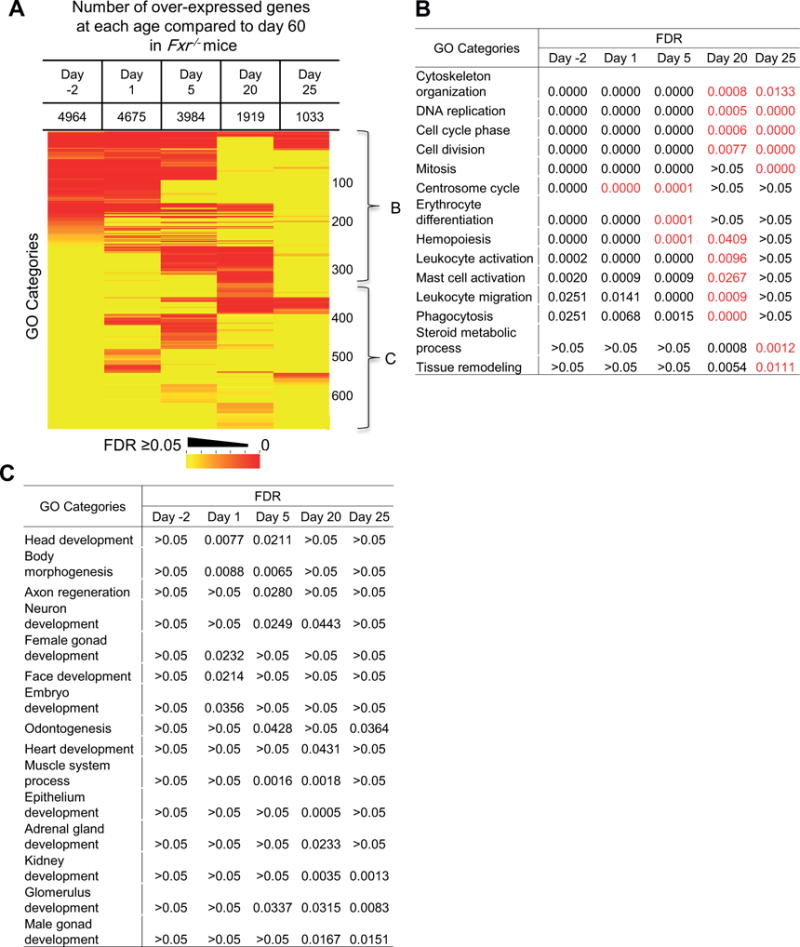
Gene ontology analysis for over-expressed genes during postnatal liver maturation compared to day 60 in Fxr^−/−^ mice. (A) Heat map for GO categories with significant gene over-expression at ages of day −2, 1, 5, 20, and 25. The color represents the false discovery rate of each GO category. The numbers on the right are the cumulative number of GO categories. The brackets B and C are used to label different groups of GO categories. Representative GO categories in each bracket are show in the corresponding panels of (B) and (C). The FDR values with red color in panel (B) indicate the same GO categories having a FDR > 0.05 in the age-matched C57BL/6 samples.

**Figure 6:**
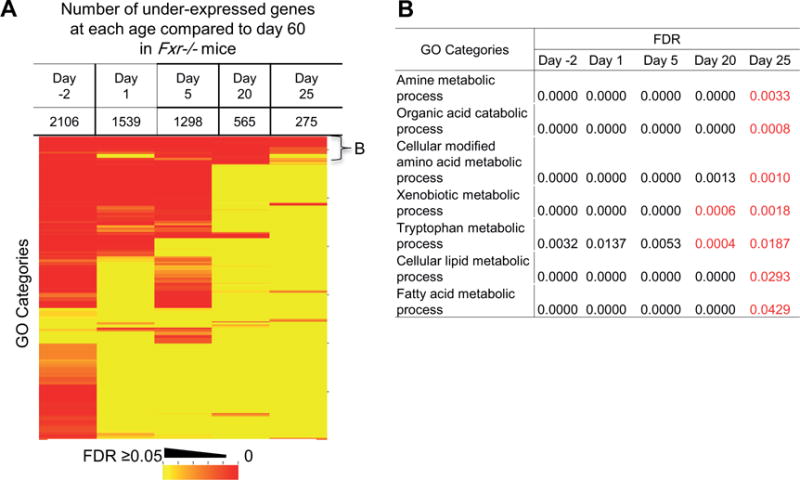
Gene ontology analysis for under-expressed genes during postnatal liver maturation compared to day 60 in Fxr^−/−^ mice. (A) Heat map for GO categories with significant gene under-expression at ages of day −2, 1, 5, 20, and 25. The color represents the false discovery rate of each GO category. The numbers on the right are the cumulative number of GO categories. The bracket B is used to label a group of GO categories. (B) Representative GO categories in the bracket B. The FDR values with red color indicate the same GO categories having a FDR > 0.05 in the age-matched C57BL/6 samples.

At 20- and 25-days of age compared to 60-days of age, there were more differentially expressed genes in *Fxr*^−/−^ samples than in C57BL/6 samples (Figure 5A and 6A). The enriched GO categories for over-expressed genes in livers of *Fxr*^−/−^ mice could generally be divided into two groups (bracket B and C in Figure 5A). One group contained similar GO categories, such as cell cycle, cellular processes, and hematopoiesis that exhibited in the analysis of C57BL/6 samples, but they were not as age-specific as in C57BL/6 and enriched at later ages in *Fxr*^−/−^ samples (Figure 5B). The other group contained GO categories not appeared in C57BL/6 samples, and they were mainly pathways for other organ development instead of the liver, such as head, neuron, heart, muscle, epithelium, adrenal gland, kidney, and male gonad (Figure 5C). This result indicted that the nuclear receptor FXR might have important roles to maintain the liver identity during postnatal development. The enriched GO categories for under-expressed genes in *Fxr*^−/−^samples were similar to that in C57BL/6 samples, including numerous metabolic processes, such as xenobiotics, lipid, fatty acids, and amino acids (Figure 6B). But many of them displayed delayed maturation and were continually enriched at later ages.

### 3.5. Effects of *Fxr*^−/−^ on the expression of hepatic nuclear receptors and core transcription factors

**Figure 7:**
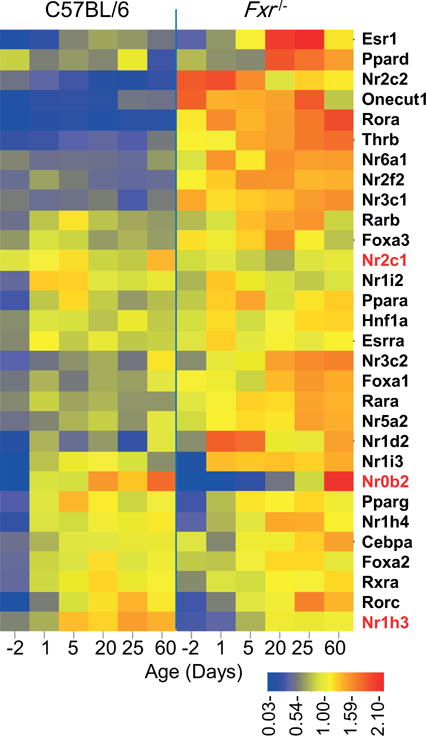
The effect of Fxr^−/−^ on ontogenic expression of nuclear receptors and core transcription factors in liver. Heat map of expression profiles are drawn for all nuclear receptors and main hepatic transcription factors (genes labeled on the right) with significant differential expression between C57BL/6 and Fxr^−/−^ samples. For each gene, the value of log_2_(1+[fold change of mean FPKM]) over the ages are calculated to show the trends of expression. The three gene names in red color have higher expression in C57BL/6 samples than in Fxr^−/−^ samples.

Previous studies on the regulation of liver-specific genes have identified a core group of hepatic regulators, which form a complex hepatic transcription factor network during liver development [28]. To assess the role of FXR in this network and the effect of *Fxr*^−/−^ on the expression of hepatic transcription regulators, we examined the developmental expression of core transcription factors and all nuclear receptor family members in livers of C57BL/6 and *Fxr*^−/−^ mice. A total of 53 genes in the transcription regulator group were examined (Supplemental Table S4 available online at http://www.agialpress.com/journals/nurr/2017/101308). Among them, 14 genes were not expressed in liver, including *Nr2e1*, *Rorb*, *Nr0b1*, *Nr2e3*, *Esr2*, *Nr5a1*, *Nr4a3*, *Nr4a2*, *Vdr*, *Hnf4g*, *Esrrb*, *Nr2f1*, *Esrrg*, and *Nr4a1.* Other 9 gene were expressed in liver but had no significant changes between C57BL/6 and *Fxr*^−/−^ samples, which are *Rxrg*, *Rarg*, *Hnf1b*, *Thra*, *Nr1d1*, *Rxrb*, *Nr1h2*, *Nr2f6*, and *Hnf4a.* The remaining 30 genes had significant differential expression between C57BL/6 and *Fxr*^−/−^ mice for at least one age during postnatal liver maturation (Figure 7). Within the differentially expressed genes, only 3 showed decreased expression in the *Fxr*^−/−^ samples, including *Nr2c1*, *Nr1h3*, and the known FXR target gene *Nr0b2*, and the rest 27 genes all increased in the *Fxr*^−/−^ samples. The increased expression of most hepatic transcription regulators in *Fxr*^−/−^ mice was probably a result of compensatory effects for the loss of FXR, and suggested widespread interactions within the transcription regulatory network, where regulators cooperated in determining the hepatic functions.

## 4. Discussion

In this study, we used RNA-Seq to examine alteration of transcriptome in liver during postnatal maturation in *Fxr*^−/−^ and its control background C57BL/6 mice that resulted in two major findings: (1) the timeline of liver functional transition during postnatal maturation; and (2) novel roles of FXR in control of liver postnatal maturation.

Liver as the largest vital organ in the body is completely formed at birth, but not fully mature. During postnatal maturation, many genes in the liver alter their expression to achieve functional transition from a prenatal hematopoiesis organ to an adult metabolic organ. By using RNA-Seq, we detected approximately 45% of the 23,196 annotated mouse genes were transcribed in the prenatal liver at day −2. Transcribed genes were decreased to 36% in the matured liver at day 60 (Table 1). Pearson correlation coefficient *r* value between livers on day −2 and day 60 is less than 0.1 (Figure 2), indicating the functions as reflected by gene transcription levels are significantly altered from prenatal to adult livers. A gradual transition occurs during the postnatal liver maturation. Therefore, we used the transcriptome at day 60 as a mature reference liver to compare to the transcriptomes at each younger age, representing prenatal, neonatal, and adolescent livers. The GO pathway analysis of the differentially expressed genes demonstrated that most of the highly expressed genes at early ages belonged to diverse biological processes of cell proliferation, basic cellular metabolism, cellular structure assembly, and hematopoiesis (Figure 3). However, with increase of age, the liver cells became more static and functionally more specialized in metabolism (Figure 4). Thus the expressed genes changed to a more concentrated group.

Our GO analysis revealed the details of biological changes that happened during postnatal liver maturation. During the perinatal stage (day −2 to 5), the liver underwent rapid cell proliferation and growth and blood cell development was still active in liver (Figure 3B). During the neonatal stage (day 1 and 5), there was extensive immune cell activation as the newborns were exposed to the environment and started food intake, which introduced enormous challenges to the immune system. But the immunity in liver was not matured at this stage (Figure 3C), which may explain the common diagnosis of sepsis in neonates [29]. At day 20, the liver went through changes of cell adhesion and tissue structure maturation (Figure 3D). Some metabolic pathways were over-expressed at this age, and this might correspond to the cause of increased resistance to certain drugs in young children [30]. Genes in most metabolic processes were under-expressed at young ages (Figure 4). The ones that matured at earlier ages were in pathways for the metabolism of the most critical nutrients, like glucose, lipids, and amino acids (Figure 4D). The critical function of blood coagulation also matured immediately after birth to prevent life-threatening hemorrhage. As the mice were usually weaned and separated from the mother three weeks after birth, this was the time that most of the liver functions became mature. At day 25, very few genes were still differentially expressed compared to day 60.

FXR is selected in this study to examine its roles in control of biological processes during postnatal liver maturation because FXR has been identified as a key regulator of functions in a mature liver, such as maintaining homeostasis of bile acids, lipids, and glucose [31]. Bile acids are immediately produced by the liver after birth to help absorption of the mother’s milk and act as endogenous signals [14]. RNA-Seq in this study also revealed an immediate increase of FXR transcription from prenatal to neonatal livers, and then FXR was expressed at a consistent level throughout postnatal maturation (Figure 1). By deletion of the last exon of the *Fxr* gene, *Fxr*^−/−^mice have served as a powerful tool to study the roles of FXR in liver functions [25, 26, 32]. These knockout mice still express a truncated form of the nuclear receptor that lacks the functional ability to recognize ligands. However, it is important to note that the protein still contains the DNA binding domain and may still have constitutive activity even in the absence of ligand activation. Therefore, in this study, we used *Fxr*^−/−^mice to determine the role of FXR activation in control of gene transcription during postnatal liver maturation. In comparison of the Pearson correlation coefficient *r* values across the different developmental ages, a clear dissimilarity of transcriptome between each developmental stage of prenatal, neonatal, adolescent, and adult in C57BL/6 mice was diminished in the *Fxr*^−/−^mice, especially during the neonatal (day 1) and adolescent (day 25) ages (*r* value is appropriate 0.8 in *Fxr*^−/−^vs. 0.6 in C57BL/6 mice in Figure 2), indicating that without FXR, liver maturation process is delayed. The same conclusion can also be obtained from the GO analysis. The effect of FXR on postnatal liver maturation was most significant at the adolescent stage, as the pathways specifically active during the neonatal stage in C57BL/6 mice failed to be shut down at adolescent stage in *Fxr*^−/−^mice, and the pathways that should mature before the adolescent stage remained immature in *Fxr*^−/−^ mice (Figure 5 and 6). The surge of bile acids during this period, as shown by previous studies [14], may underlie the critical function of FXR at this developmental stage.

FXR may interact with other key transcriptional regulators to achieve its roles in control of liver functions. Six hepatic transcription factors have been identified to form complex autoregulatory and cross-regulatory circuits [28]. Through analysis of genome-wide FXR binding sites, we have provided evidence to show that FXR is involved in broad biological pathways in maintaining hepatic homeostasis [33], and FXR co-regulates gene transcription with HNF4 in mouse liver on a genome-wide scale [34]. In this study, our result of the multiple organ developmental processes in *Fxr*^−/−^ mice further confirmed the role of FXR as a core hepatic nuclear receptor that defines the hepatic phenotype. And this may be a more pervasive phenomenon as most of the nuclear receptors together with the core transcription factors showed altered expressions in *Fxr*^−/−^ mice. While most transcriptional regulators displayed increased expression, probably to compensate FXR function, the liver X receptor α (LXRα, Nr1h3) showed significantly decreased expression at all ages. Interestingly, LXRs and FXR are known to be the yin and yang of cholesterol and fat metabolism, maintaining a balanced regulation of cholesterol and bile acid metabolism [35]. And our results suggest a further interplay between these two nuclear receptors, as FXR may be involved in the regulation of LXRα.

Postnatal liver development is largely an under-studied area. Some groups have identified certain critical factors for postnatal liver development, including β-Catenin and Yes-associated protein [36, 37]. These two factors showed increased expression during postnatal liver development to promote cell proliferation, and deletion of either of them might lead to impaired liver growth. Our transcriptome data also revealed the developmental expression of β-Catenin and Yes-associated protein. They were both induced in *Fxr*^−/−^ mice, which is consistent with the prolonged cell proliferation in *Fxr*^−/−^ livers. And the result further indicates their direct roles in control of liver growth.

Quantifying the developmental transcriptome is an initial step to study postnatal liver maturation. It utilized advanced technology to generate an overall picture of gene expression, and revealed potential fields of interest that await further research. For example, histology and liver functional studies based on the transcriptome data would help provide a more defined concept of how the liver matures. This study can also give greater insight into the role nutrition may play in liver development. Decreases in FXR signaling due to lowered fat intake and decreases in bile acid production may alter hepatic gene expression ontogeny. Future studies to over-express FXR or treat animals with FXR agonists during development may complement the loss-of-function study and help differentiate the direct and indirect effects of FXR in regulating postnatal liver development. Altogether, these studies would enable a more profound understanding of liver development and assist in the management of diseases in liver.

## Supplementary Material

Supplemental Figure 1

Supplemental Table 1

Supplemental Table 2

Supplemental Table 3

Supplemental Table 4

## Figures and Tables

**Table 1:** Number of gene expressed in liver at different ages.

Developmental age	Day −2	Day 1	Day 5	Day 20	Day 25	Day 60
No genes expressed in C57BL/6	10,338	10,534	10,402	8,801	8,488	8,507
No. genes expressed in *Fxr*^−/−^	11,557	11,708	11,698	10,873	10,696	10,058
