# Supplementary material for: Role of Farnesoid X Receptor in the Determination of Liver Transcriptome during Postnatal Maturation in Mice
Source: Nucl Receptor Res. Author manuscript; Available in PMC 2018 May 21. (PMC5962295; doi:10.11131/2017/101308)
Supplement: Supplemental Figure 1 [file NIHMS934907-supplement-Supplemental_Figure_1.pdf]

Supplemental Figure S1.

RNA-seq and RT-PCR validation results for selected genes at different ages in wild-type and *Fxr*$^{-/-}$ mice.

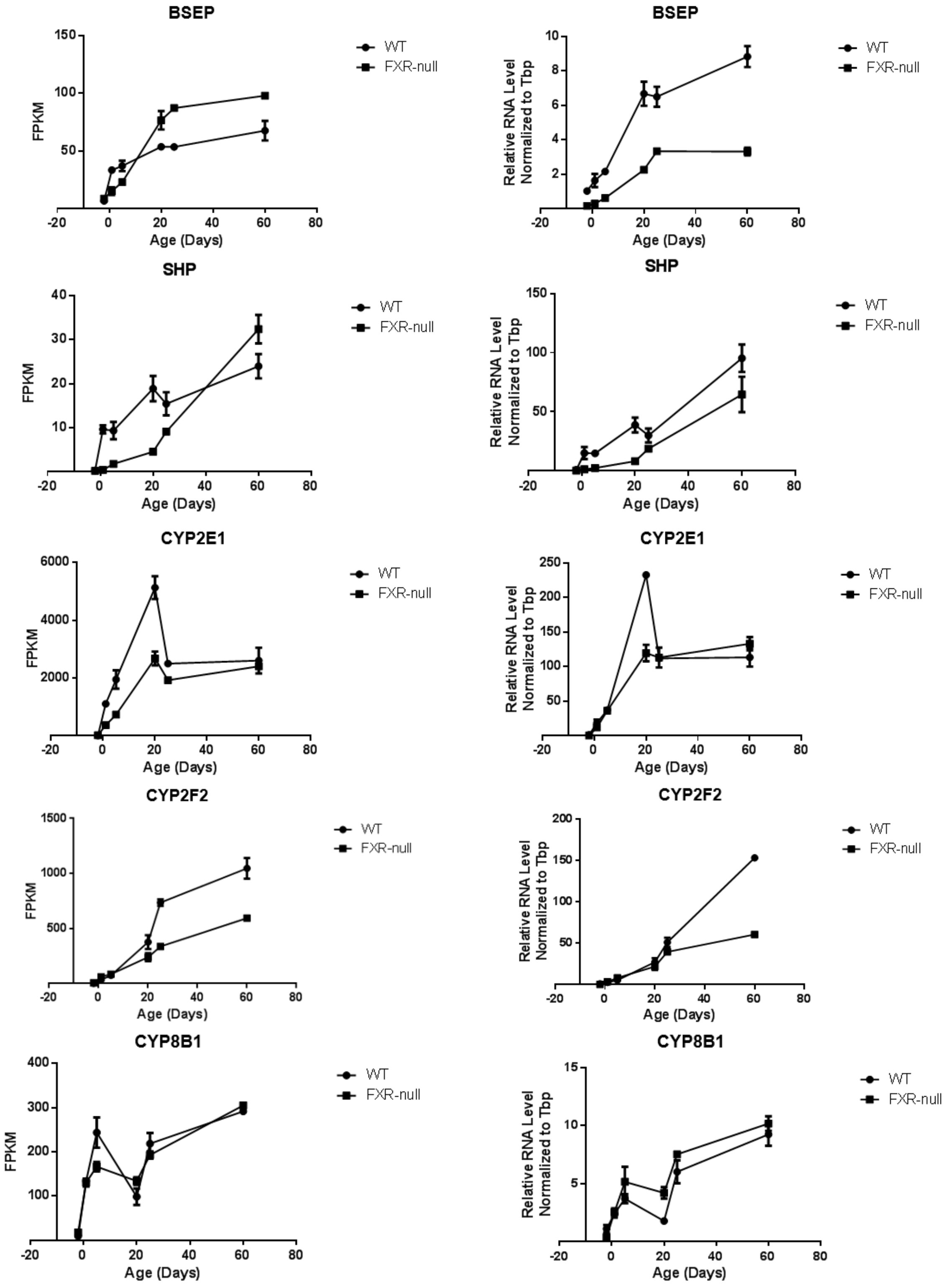

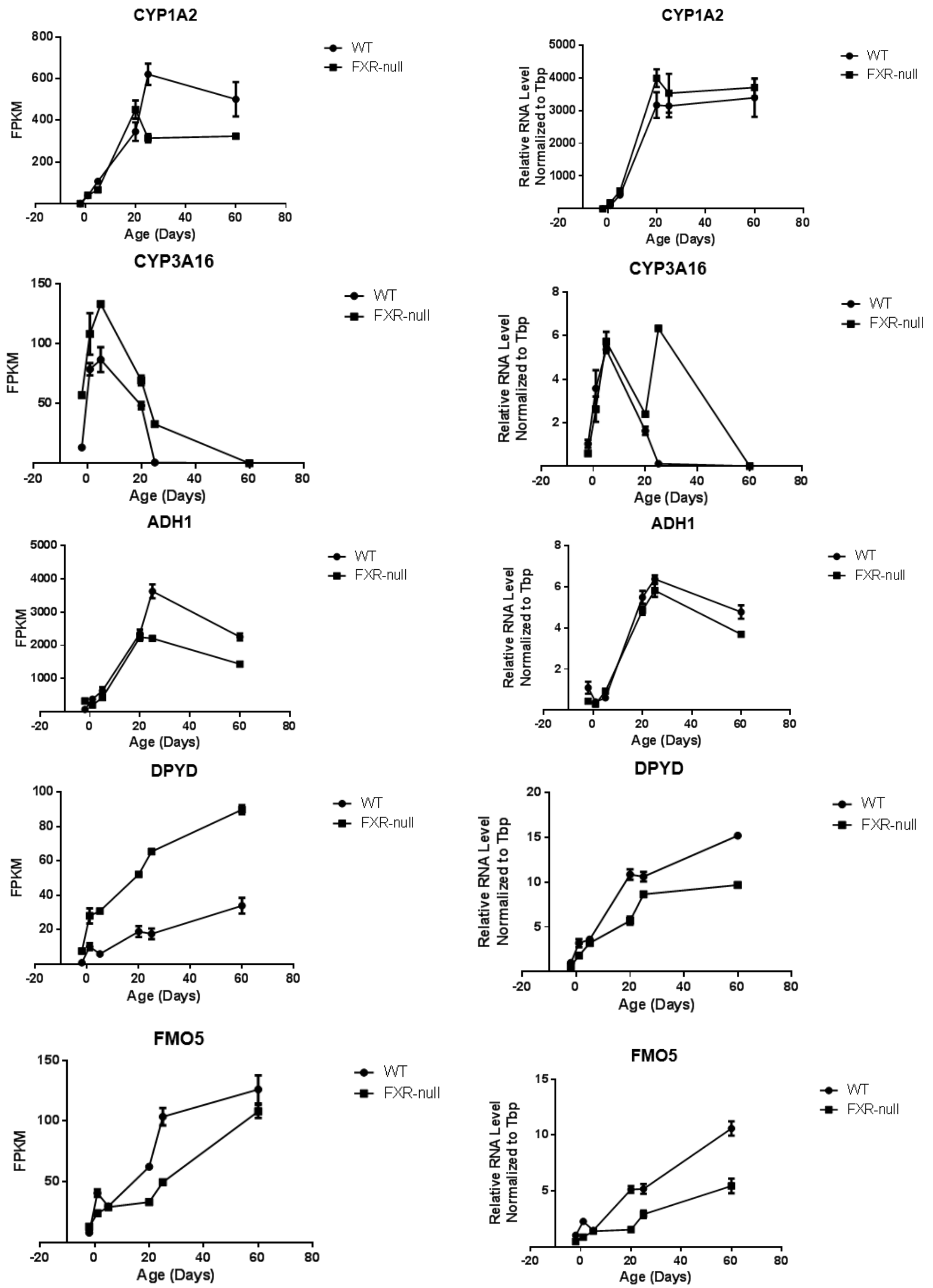
CYP1A2
FPKM
Age (Days)
WT
FXR-null
CYP1A2
Relative RNA Level Normalized to Tbp
Age (Days)
WT
FXR-null
CYP3A16
FPKM
Age (Days)
WT
FXR-null
CYP3A16
Relative RNA Level Normalized to Tbp
Age (Days)
WT
FXR-null
ADH1
FPKM
Age (Days)
WT
FXR-null
ADH1
Relative RNA Level Normalized to Tbp
Age (Days)
WT
FXR-null
DPYD
FPKM
Age (Days)
WT
FXR-null
DPYD
Relative RNA Level Normalized to Tbp
Age (Days)
WT
FXR-null
FMO5
FPKM
Age (Days)
WT
FXR-null
FMO5
Relative RNA Level Normalized to Tbp
Age (Days)
WT
FXR-null
