# Supplementary material for: Role of Farnesoid X Receptor in the Determination of Liver Transcriptome during Postnatal Maturation in Mice
Source: Nucl Receptor Res. Author manuscript; Available in PMC 2018 May 21. (PMC5962295; doi:10.11131/2017/101308)
Supplement: Supplemental Table 1 [file NIHMS934907-supplement-Supplemental_Table_1.pdf]

Supplemental Table S1.

Forward and reverse primer sequences for RT-PCR validation of selected genes.

Gene	Forward	Reverse
Bsep	GAGGCGACAATGGAAGACATA	CTTCTCCAACTAGGGTGTCAAA
Shp	GCACTGTGTGAAGTCTTGGA	CTGGAATGTTCTTGAGGGTAGAG
Cyp2e1	CACAGCCAAGAACCCATGTA	CATGAGAATCAGGAGCCCATATC
Cyp2f2	CGACTGCTTCCTCACAAAGA	GTCATCAGCAGGGTATCCATATT
Cyp8b1	TTTCTGAGGGAGCAAGGAATAG	GGAATAAGAGGACCCAGAAACA
Cyp1a2	CAAGCACAGCGAGAACTACAA	GTCAAAGCCAGCTCCAAAGA
Cyp3a16	GAGATCACAGCCCAGTCAATTA	GTGAGTGGCCAAGGAATACA
Adh1	GAAGAAGTCTACAAGGACCCATC	CACCGCAGCTTTGCATTT
Dpyd	CCTGGACAAAGCTCCTTTCTTA	GATGCTGGCGATCAGGATATT
Fmo5	GAAGGCAGGGCCAGTATTT	TGGGAGTTGTGCATGTAGTTAG
