# Supplementary material for: Role of Farnesoid X Receptor in the Determination of Liver Transcriptome during Postnatal Maturation in Mice
Source: Nucl Receptor Res. Author manuscript; Available in PMC 2018 May 21. (PMC5962295; doi:10.11131/2017/101308)
Supplement: Supplemental Table 2 [file NIHMS934907-supplement-Supplemental_Table_2.pdf]

Supplemental Table S2.

Pearson's correlation *r* value of ontogenic patterns of selected genes defined by RNA-Seq and RT-PCR in C57BL/6 and *Fxr*$^{-/-}$mice

	r value	
Gene	C57BL/6	*Fxr*$^{-/-}$
Bsep	0.947	0.993
Shp	0.889	0.999
Cyp2e1	0.969	0.984
Cyp2f2	0.929	0.996
Cyp8b1	0.857	0.973
Cyp1a2	0.936	0.984
Cyp3a11	0.964	0.960
Adh1	0.960	0.991
Dpyd	0.967	0.983
Fmo5	0.922	0.996

Correlation of ontogenic patterns of gene expression was performed between the data generated by RNA-Seq and RT-PCR of C57BL/6 and *Fxr*$^{-/-}$mice.
