# Supplementary material for: Role of Farnesoid X Receptor in the Determination of Liver Transcriptome during Postnatal Maturation in Mice
Source: Nucl Receptor Res. Author manuscript; Available in PMC 2018 May 21. (PMC5962295; doi:10.11131/2017/101308)
Supplement: Supplemental Table 4 [file NIHMS934907-supplement-Supplemental_Table_4.pdf]

**Supplemental Table S4**.
The list of hepatic transcription regulators.

| Gene | Status |
|---|---|
| Esr1 | Increased in *Fxr-/-* samples |
| Rora | Increased in *Fxr-/-* samples |
| Nr3c2 | Increased in *Fxr-/-* samples |
| Nr1d2 | Increased in *Fxr-/-* samples |
| Onecut1 | Increased in *Fxr-/-* samples |
| Thrb | Increased in *Fxr-/-* samples |
| Nr6a1 | Increased in *Fxr-/-* samples |
| Nr1i3 | Increased in *Fxr-/-* samples |
| Rarb | Increased in *Fxr-/-* samples |
| Nr2c2 | Increased in *Fxr-/-* samples |
| Rorc | Increased in *Fxr-/-* samples |
| Nr2f2 | Increased in *Fxr-/-* samples |
| Foxa1 | Increased in *Fxr-/-* samples |
| Pparg | Increased in *Fxr-/-* samples |
| Rara | Increased in *Fxr-/-* samples |
| Nr5a2 | Increased in *Fxr-/-* samples |
| Nr1h4 | Increased in *Fxr-/-* samples |
| Foxa2 | Increased in *Fxr-/-* samples |
| Nr3c1 | Increased in *Fxr-/-* samples |
| Hnf1a | Increased in *Fxr-/-* samples |
| Esrra | Increased in *Fxr-/-* samples |
| Ppard | Increased in *Fxr-/-* samples |
| Ppara | Increased in *Fxr-/-* samples |
| Nr1i2 | Increased in *Fxr-/-* samples |
| Foxa3 | Increased in *Fxr-/-* samples |
| Rxra | Increased in *Fxr-/-* samples |
| Cebpa | Increased in *Fxr-/-* samples |
| Nr0b2 | Decreased in *Fxr-/-* samples |
| Nr2c1 | Decreased in *Fxr-/-* samples |
| Nr1h3 | Decreased in *Fxr-/-* samples |
| Rxrg | Not significantly changed between wild-type and *Fxr-/-* samples |
| Rarg | Not significantly changed between wild-type and *Fxr-/-* samples |
| Hnf1b | Not significantly changed between wild-type and *Fxr-/-* samples |
| Thra | Not significantly changed between wild-type and *Fxr-/-* samples |
| Nr1d1 | Not significantly changed between wild-type and *Fxr-/-* samples |
| Rxrb | Not significantly changed between wild-type and *Fxr-/-* samples |
| Nr1h2 | Not significantly changed between wild-type and *Fxr-/-* samples |
| Nr2f6 | Not significantly changed between wild-type and *Fxr-/-* samples |

| | |
|---|---|
| Hnf4a | Not significantly changed between wild-type and *Fxr-/-* samples |
| Nr2e1 | Not expressed in liver |
| Rorb | Not expressed in liver |
| Nr0b1 | Not expressed in liver |
| Nr2e3 | Not expressed in liver |
| Esr2 | Not expressed in liver |
| Nr5a1 | Not expressed in liver |
| Nr4a3 | Not expressed in liver |
| Nr4a2 | Not expressed in liver |
| Vdr | Not expressed in liver |
| Hnf4g | Not expressed in liver |
| Esrrb | Not expressed in liver |
| Nr2f1 | Not expressed in liver |
| Esrrg | Not expressed in liver |
| Nr4a1 | Not expressed in liver |
